# Supplementary material for: A synthetic PGC1α co-activator reprograms microglial immunometabolism to preserve cone function in retinal degeneration
Source: bioRxiv. 2026 Oct 5:2026.09.29.753881. Preprint. [Version 1] doi: 10.64898/2026.09.29.753881 (PMC13691950; doi:10.64898/2026.09.29.753881)
Supplement: Supplementary file 1 [file NIHPP2026.09.29.753881V1-supplement-1.pdf]

bioRxiv preprint doi: https://doi.org/10.64898/2026.09.29.753881; this version posted October 5, 2026. The copyright holder for this preprint (which was not certified by peer review) is the author/funder, who has granted bioRxiv a license to display the preprint in perpetuity. It is made available under a CC-BY 4.0 International license.

A synthetic PGC1α co-activator reprograms microglial immunometabolism to preserve cone function in retinal degeneration

Thomas Winogrodzki[1], Jayvin Hernandez-Reyes[1], Rajvir Solanky[1], Richard Lowery[2], Georgy Komissarov[1], Wen Huang[1], Boyuan Wang[1,3], Neoklis Makrides[1], Sharifa Davis[1,3], Siyuan Liu[1,3], Yong-Shi Li[1], Wen-Hsuan Wu[1], Nicholas Nolan[1], Robert van de Werken[1,3], Aykut Demirkol[1], Anders Knudsen[1,3], Nuntachai Surawatsatien[1,4], Yao Li[1], James Hurley[2], Stephen H Tsang[1]

[1]Department of Ophthalmology, Columbia University, New York, NY, United States

[2]Department of Biochemistry, University of Washington, Seattle, WA, United States

[3]Department of Biomedical Engineering, Columbia University, New York, NY, United States

[4]Center of Excellence in Retina, Department of Ophthalmology, Faculty of Medicine, Chulalongkorn University and King Chulalongkorn Memorial Hospital, Thai Red Cross Society, Bangkok, Thailand

Correspondence: Stephen Tsang; sht2@cumc.columbia.edu

Supplementary Information

bioRxiv preprint doi: https://doi.org/10.64898/2026.09.29.753881; this version posted October 5, 2026. The copyright holder for this preprint (which was not certified by peer review) is the author/funder, who has granted bioRxiv a license to display the preprint in perpetuity. It is made available under a CC-BY 4.0 International license.

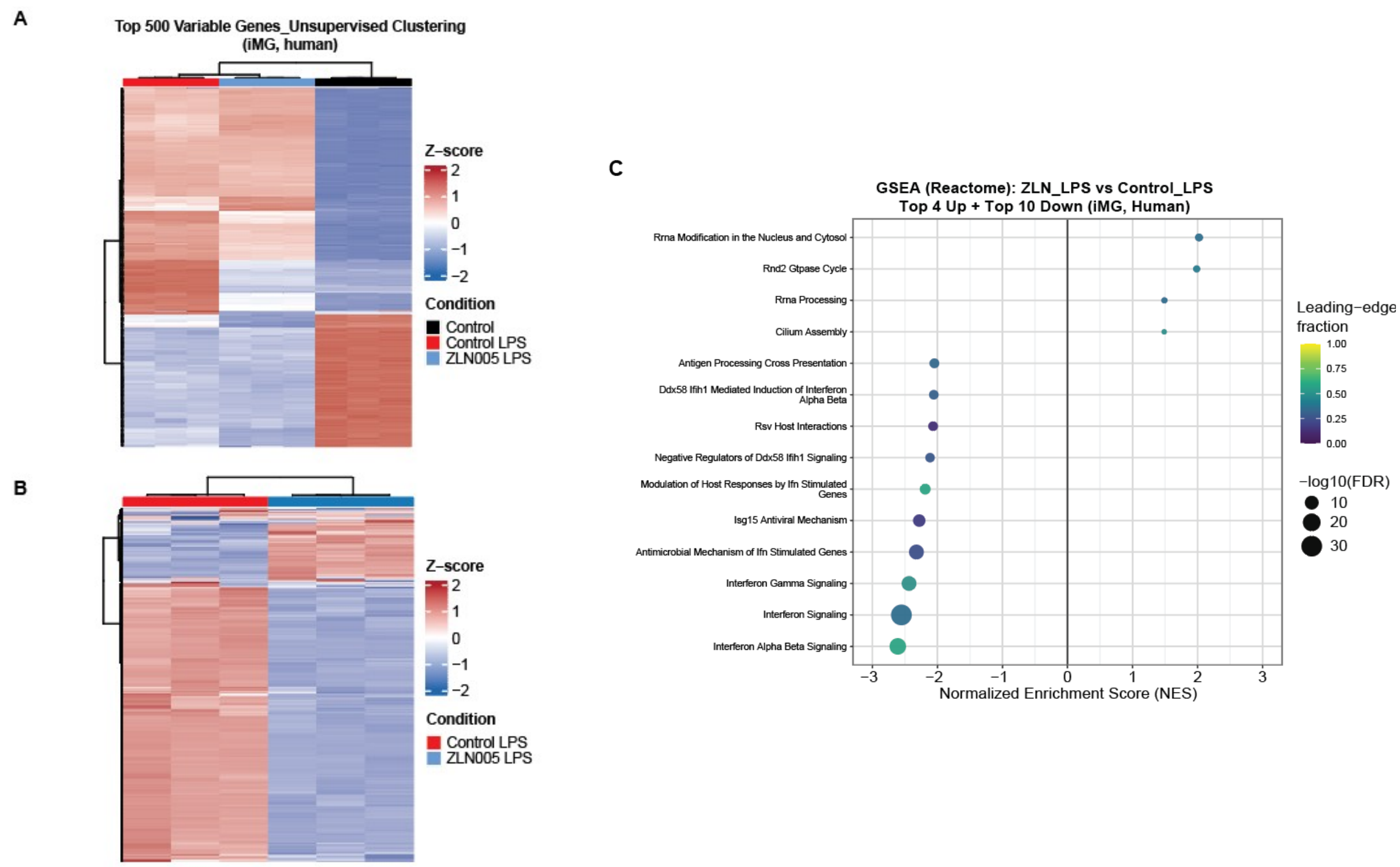

Supplementary Fig. 1. Transcriptional clustering and pathway enrichment in iPSC-derived microglia (iMG). (**A**, **B**) Unsupervised hierarchical clustering heatmaps of the top 500 most variable genes (row Z-scored): (**A**) across all three conditions (control, control+LPS, ZLN005+LPS) and (**B**) restricted to the control+LPS and ZLN005+LPS groups. (**C**) Reactome gene-set enrichment (GSEA) for the ZLN005+LPS versus control+LPS contrast (top significant 4 upregulated, top 10 downregulated pathways). The downregulated arm is dominated by interferon alpha/beta and gamma signaling and antiviral/ISG modules; positively enriched sets are non-inflammatory (rRNA processing, RND2 GTPase cycle, cilium assembly). Dot size, –log10(FDR); color, leading-edge fraction.

bioRxiv preprint doi: https://doi.org/10.64898/2026.09.29.753881; this version posted October 5, 2026. The copyright holder for this preprint (which was not certified by peer review) is the author/funder, who has granted bioRxiv a license to display the preprint in perpetuity. It is made available under a CC-BY 4.0 International license.

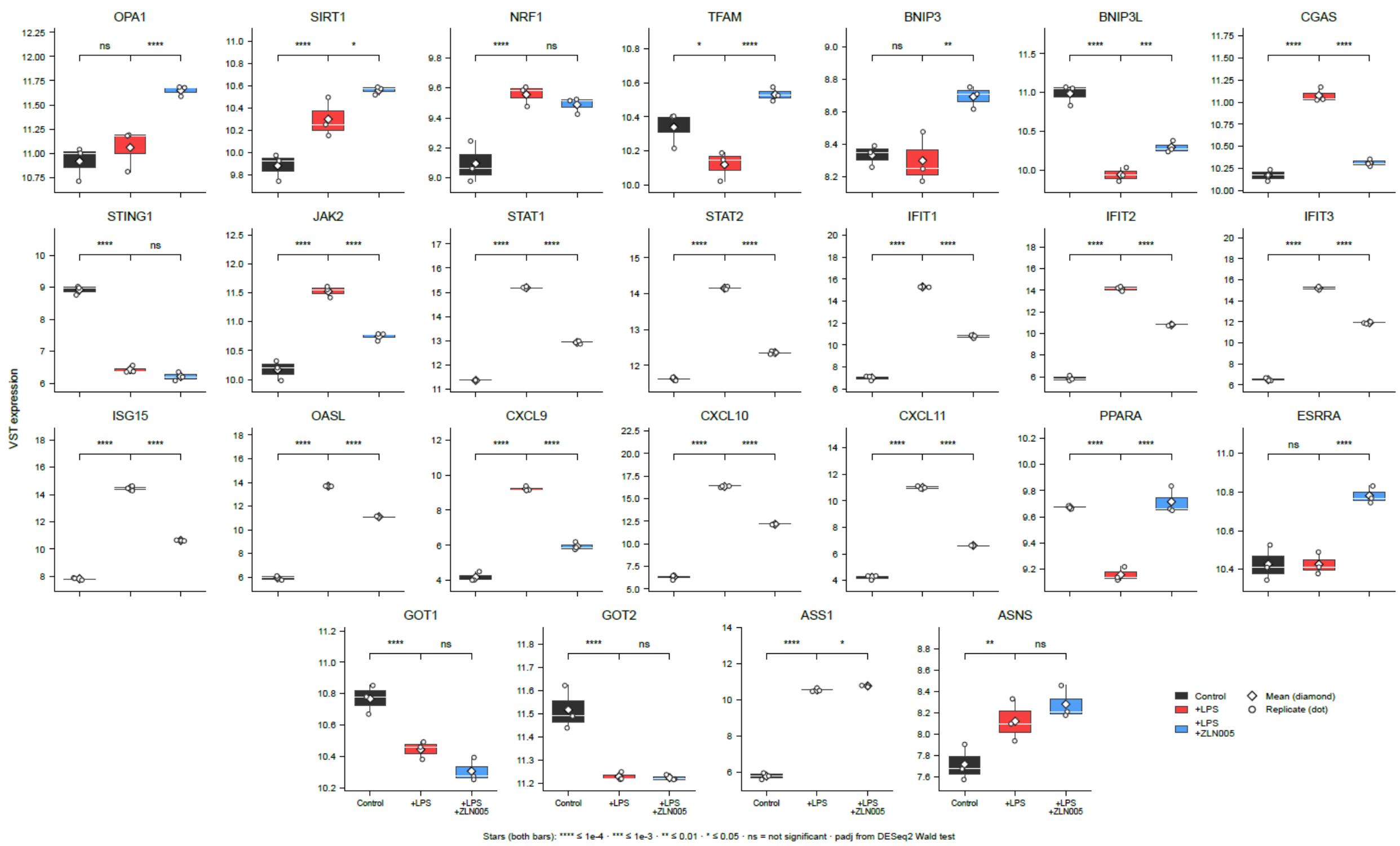

Supplementary Fig. 2. Gene-level expression of inflammatory, mitochondrial-biogenesis, and amino-acid–metabolizing transcripts in iMG. Boxplots of variance-stabilized (VST) expression for twenty-five transcripts across the three conditions (Control, +LPS, +LPS+ZLN005); boxes show the median and interquartile range, the white diamond marks the mean, and individual replicates are overlaid as points. Interferon-stimulated genes (*IFIT1, IFIT2, IFIT3, ISG15, OASL*), JAK–STAT components (J*AK2, STAT1, STAT2*) and inflammatory chemokines (*CXCL9, CXCL10, CXCL11*) are uniformly induced by LPS and significantly attenuated by ZLN005 co-treatment, whereas mitochondrial-biogenesis transcripts (*OPA1, SIRT1, NRF1, TFAM, BNIP3, BNIP3L*) show a mixed response. The cytosolic DNA sensor *CGAS*, but not STING1, is restored toward baseline by ZLN005. The PGC1α nuclear-receptor partners PPARA and *ESRRA* are induced by ZLN005 (*PPARA* is suppressed by LPS and restored toward baseline; *ESRRA* is unchanged by LPS and induced above it). Amino-acid–metabolizing enzymes are LPS-responsive: the aspartate aminotransferases *GOT1* and *GOT2* are downregulated, whereas *ASS1* and *ASNS* are upregulated, consistent with inflammatory aspartate depletion. Brackets denote the two prespecified contrasts (left, +LPS vs Control; right, +LPS+ZLN005 vs +LPS). Adjusted P values are from the DESeq2 Wald test (****$P \leq 1\times10^{-4}$; ***$P \leq 1\times10^{-3}$; **$P \leq 0.01$; *$P \leq 0.05$; ns, not significant).

bioRxiv preprint doi: https://doi.org/10.64898/2026.09.29.753881; this version posted October 5, 2026. The copyright holder for this preprint (which was not certified by peer review) is the author/funder, who has granted bioRxiv a license to display the preprint in perpetuity. It is made available under a CC-BY 4.0 International license.

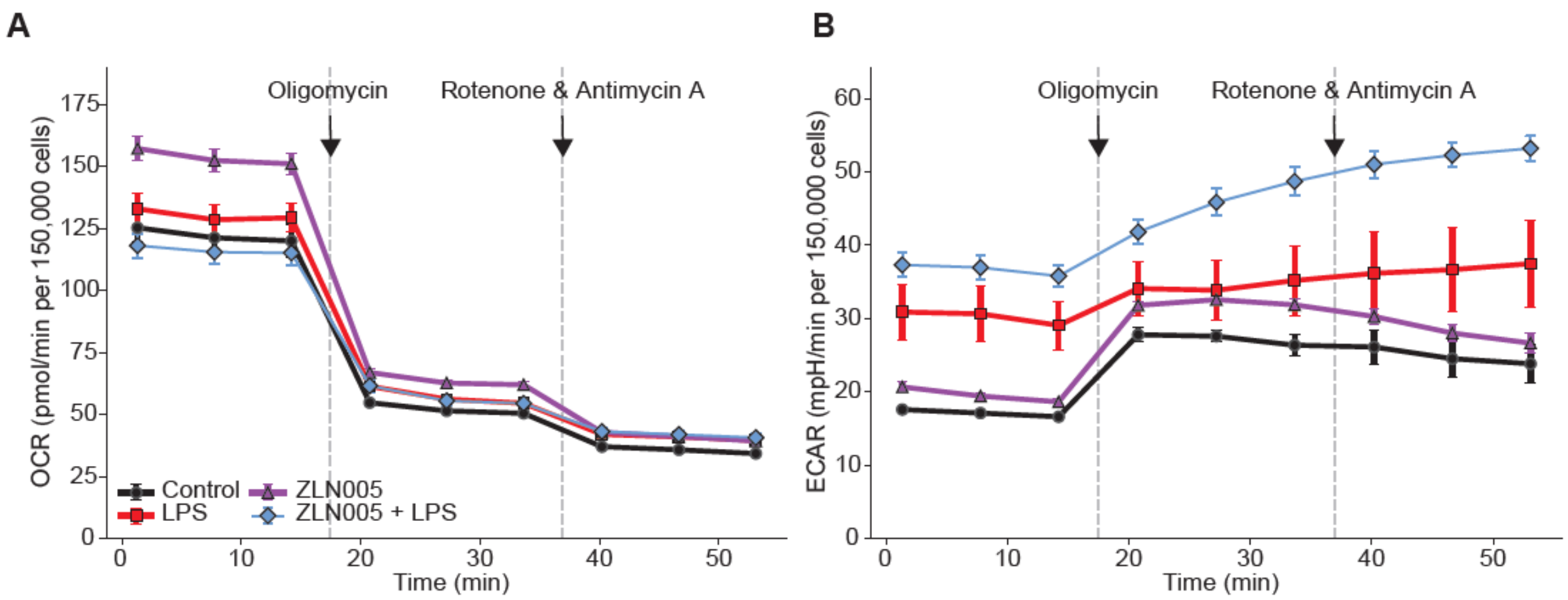

Supplementary Fig. 3. Real-time respirometry of iMG. (**A**) Oxygen consumption rate (OCR) and (**B**) extracellular acidification rate (ECAR) kinetic traces from the Seahorse ATP-rate assay across conditions, with sequential injection of oligomycin and rotenone + antimycin A (timing indicated). Cells were seeded at 150,000 cells/well and rates are expressed per 150,000 cells. n = 3 (Control, LPS) and n = 6 (ZLN005, ZLN005+LPS). Data are mean ± s.d.

bioRxiv preprint doi: https://doi.org/10.64898/2026.09.29.753881; this version posted October 5, 2026. The copyright holder for this preprint (which was not certified by peer review) is the author/funder, who has granted bioRxiv a license to display the preprint in perpetuity. It is made available under a CC-BY 4.0 International license.

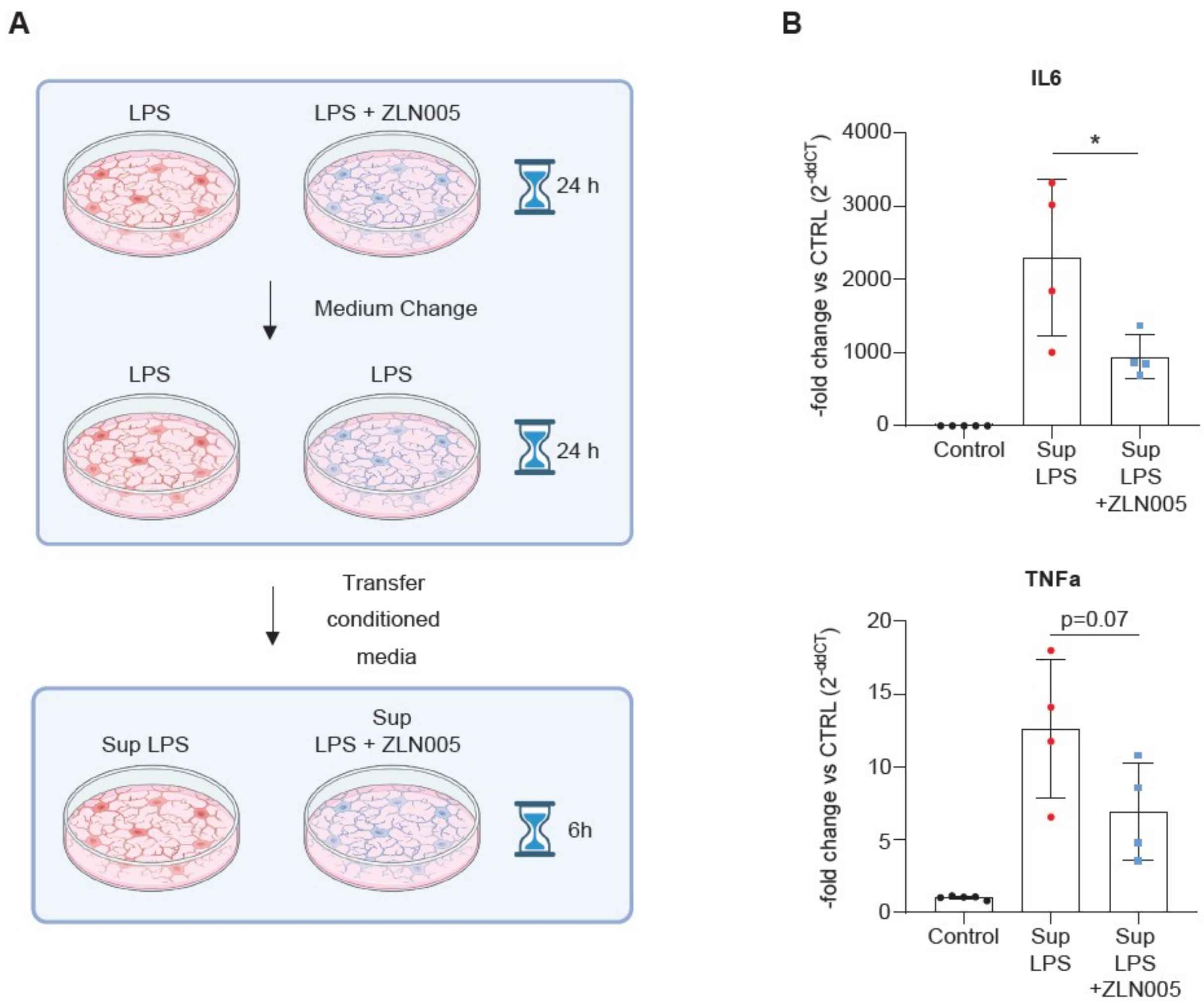

Supplementary Fig. 4. ZLN005 pre-conditioning reshapes the microglial secretome toward a less inflammatory paracrine signal. (**A**) Two-stage conditioned-medium transfer design: donor iMG were treated with LPS or LPS+ZLN005, thoroughly washed to remove ZLN005 and re-challenged with LPS alone, and their supernatants transferred to naive recipient iMG from an independent differentiation. (**B**) qPCR in recipient iMG showing reduced *IL6* induction ($P < 0.05$) and a trend toward reduced *TNFA* ($P = 0.07$) in response to the ZLN005-conditioned secretome relative to the LPS-only secretome. Expression is $2^{-\Delta\Delta Ct}$ fold-change versus untreated control; data are mean ± s.d.

bioRxiv preprint doi: https://doi.org/10.64898/2026.09.29.753881; this version posted October 5, 2026. The copyright holder for this preprint (which was not certified by peer review) is the author/funder, who has granted bioRxiv a license to display the preprint in perpetuity. It is made available under a CC-BY 4.0 International license.

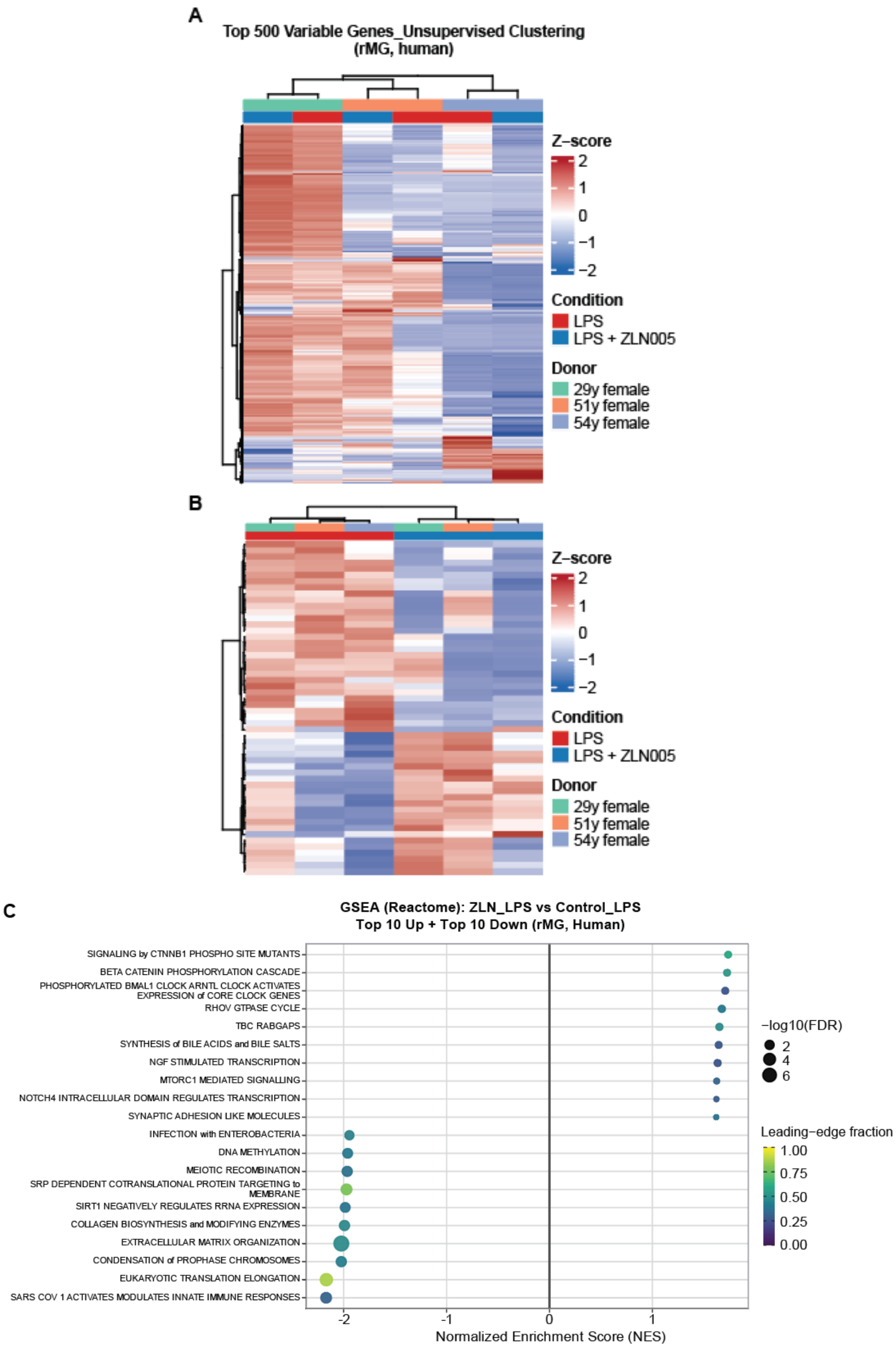

Supplementary Fig. 5. Transcriptional clustering and pathway enrichment in primary human donor retinal microglia (rMG). (**A**) Unsupervised and (**B**) supervised clustering heatmaps of the top 500 most variable genes (row Z-scored), annotated by condition (LPS, LPS+ZLN005) and donor (29, 51, 54 years, female). (**C**) Reactome GSEA for the LPS+ZLN005 versus LPS contrast (top 10 up- and top 10 downregulated pathways). ZLN005 dampens innate-immune, protein-synthesis and extracellular-matrix modules, while several anabolic/growth-associated pathways trend toward induction. Dot size, –log10(FDR); color, leading-edge fraction.

bioRxiv preprint doi: https://doi.org/10.64898/2026.09.29.753881; this version posted October 5, 2026. The copyright holder for this preprint (which was not certified by peer review) is the author/funder, who has granted bioRxiv a license to display the preprint in perpetuity. It is made available under a CC-BY 4.0 International license.

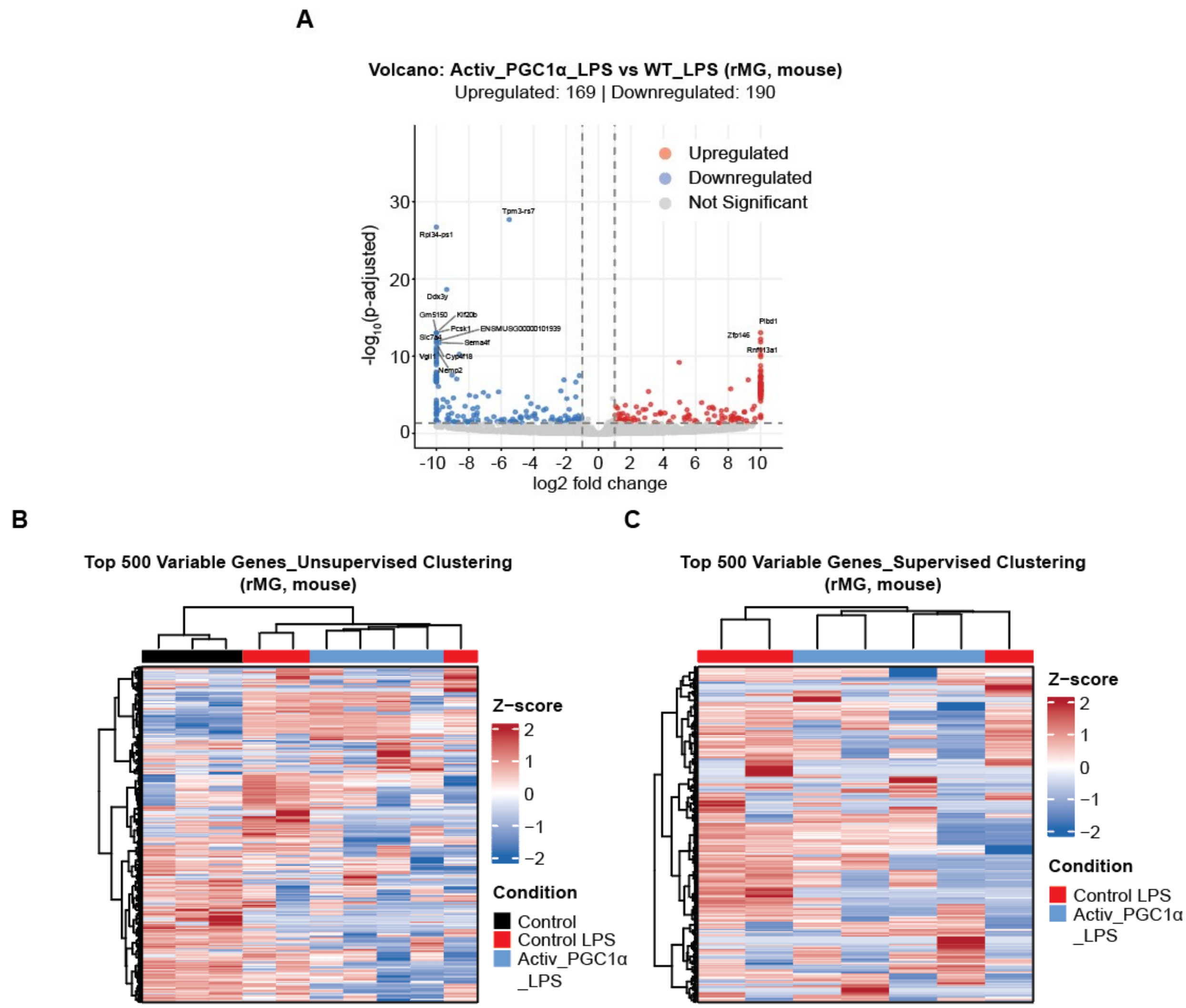

Supplementary Fig. 6. Differential expression and clustering in primary mouse retinal microglia (rMG). (**A**) Volcano plot of the Activ_PGC1α+LPS versus wildtype+LPS contrast (169 upregulated, 190 downregulated genes; DESeq2 Wald test, adjusted $P < 0.05$). (**B**, **C**) Heatmaps of the top 500 most variable genes (row Z-scored): (**B**) unsupervised clustering (control, control+LPS, Activ_PGC1α+LPS) and (**C**) supervised clustering (control+LPS versus Activ_PGC1α+LPS).

bioRxiv preprint doi: https://doi.org/10.64898/2026.09.29.753881; this version posted October 5, 2026. The copyright holder for this preprint (which was not certified by peer review) is the author/funder, who has granted bioRxiv a license to display the preprint in perpetuity. It is made available under a CC-BY 4.0 International license.

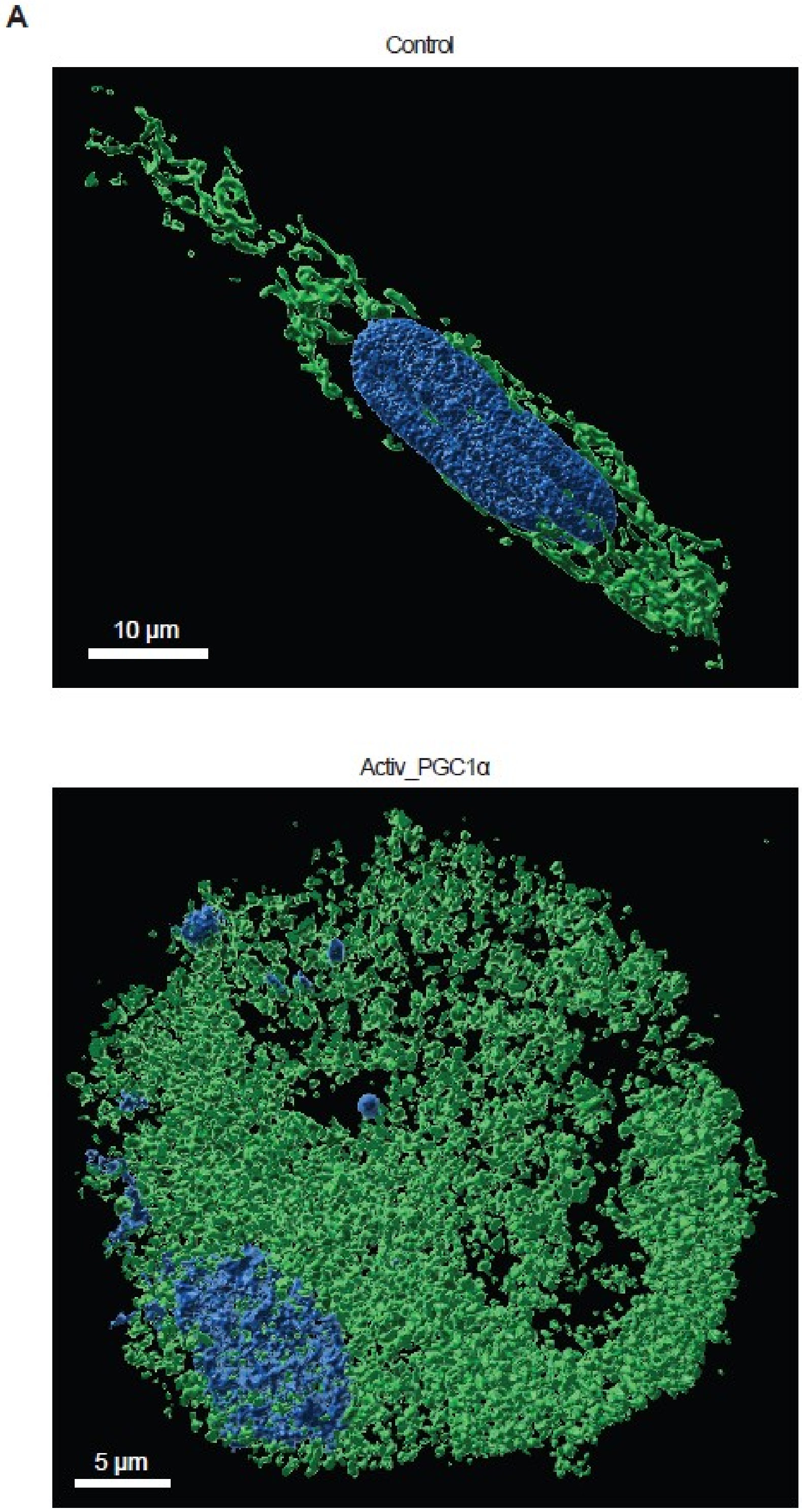

Supplementary Fig. 7. Activ_PGC1α expanded the mitochondrial network in primary retinal microglia. (**A**) Qualitative Airyscan super-resolution imaging of MitoTracker-labeled primary retinal microglia. *Activ_PGC1α*-expressing cells display an aberrant, denser mitochondrial network compared with those of wild-type controls, suggesting organelle-level confirmation of the induced mitochondrial-biogenesis program. Scale bars indicated.

bioRxiv preprint doi: https://doi.org/10.64898/2026.09.29.753881; this version posted October 5, 2026. The copyright holder for this preprint (which was not certified by peer review) is the author/funder, who has granted bioRxiv a license to display the preprint in perpetuity. It is made available under a CC-BY 4.0 International license.

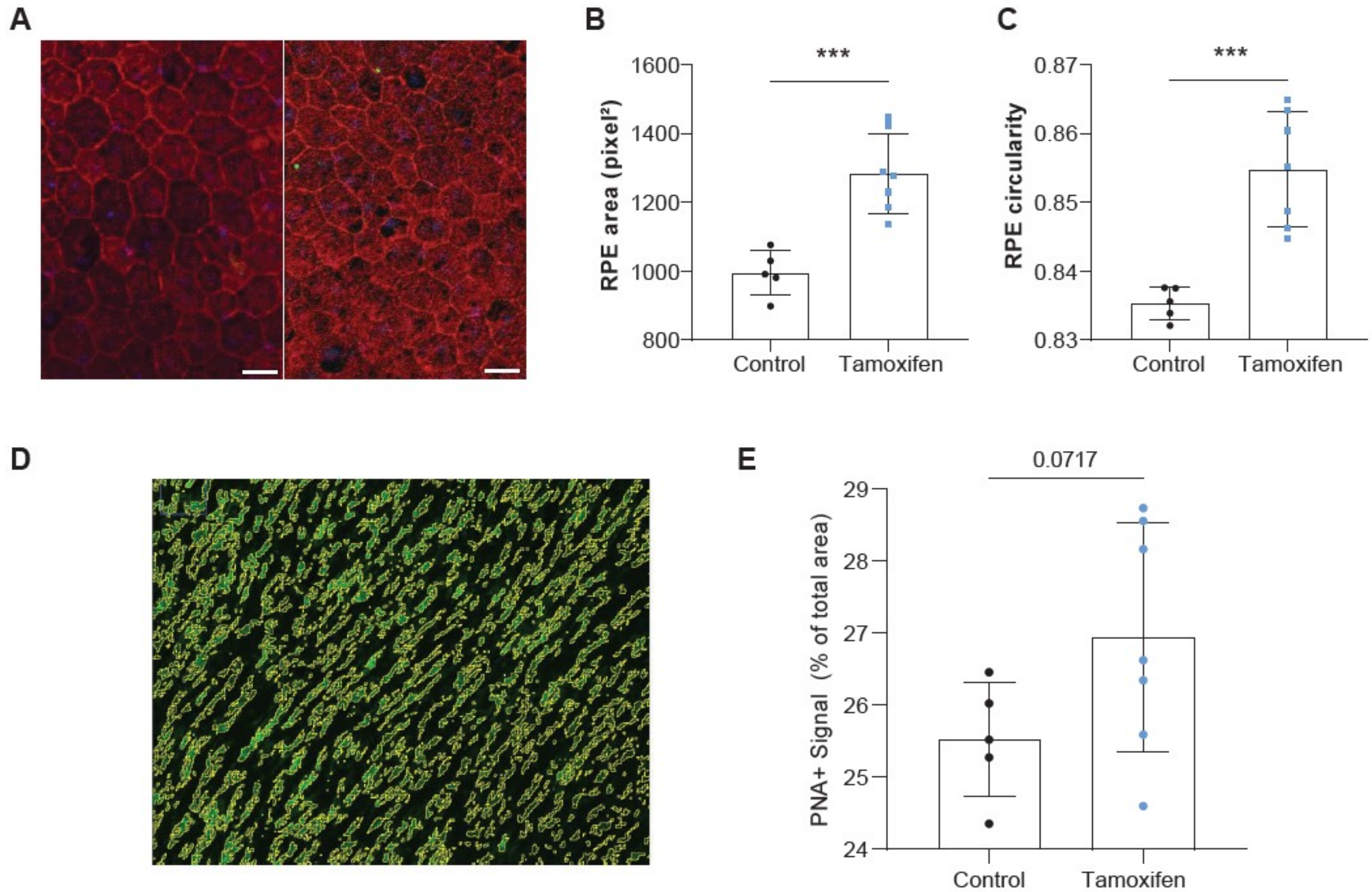

Supplementary Fig. 8. RPE and cone preservation in *Activ_PGC1α*-expressing hu*RHO*$^{P347L/+}$ retinae. (**A**) Representative RPE flatmounts stained for the tight-junction marker ZO-1 (tamoxifen - left versus control - right). Scale bar 20 µm. (**B**) RPE cell area and (**C**) RPE circularity, both significantly higher/more uniform in tamoxifen-injected animals ($P < 0.001$), indicating preservation of the regular hexagonal monolayer. (**D**) Representative PNA-labeled cone segmentation and (**E**) PNA+ cone-occupied area (% of total), showing a near-significant trend toward greater cone coverage in tamoxifen-injected animals (Welch, P = 0.072). Data are mean ± s.d

bioRxiv preprint doi: https://doi.org/10.64898/2026.09.29.753881; this version posted October 5, 2026. The copyright holder for this preprint (which was not certified by peer review) is the author/funder, who has granted bioRxiv a license to display the preprint in perpetuity. It is made available under a CC-BY 4.0 International license.

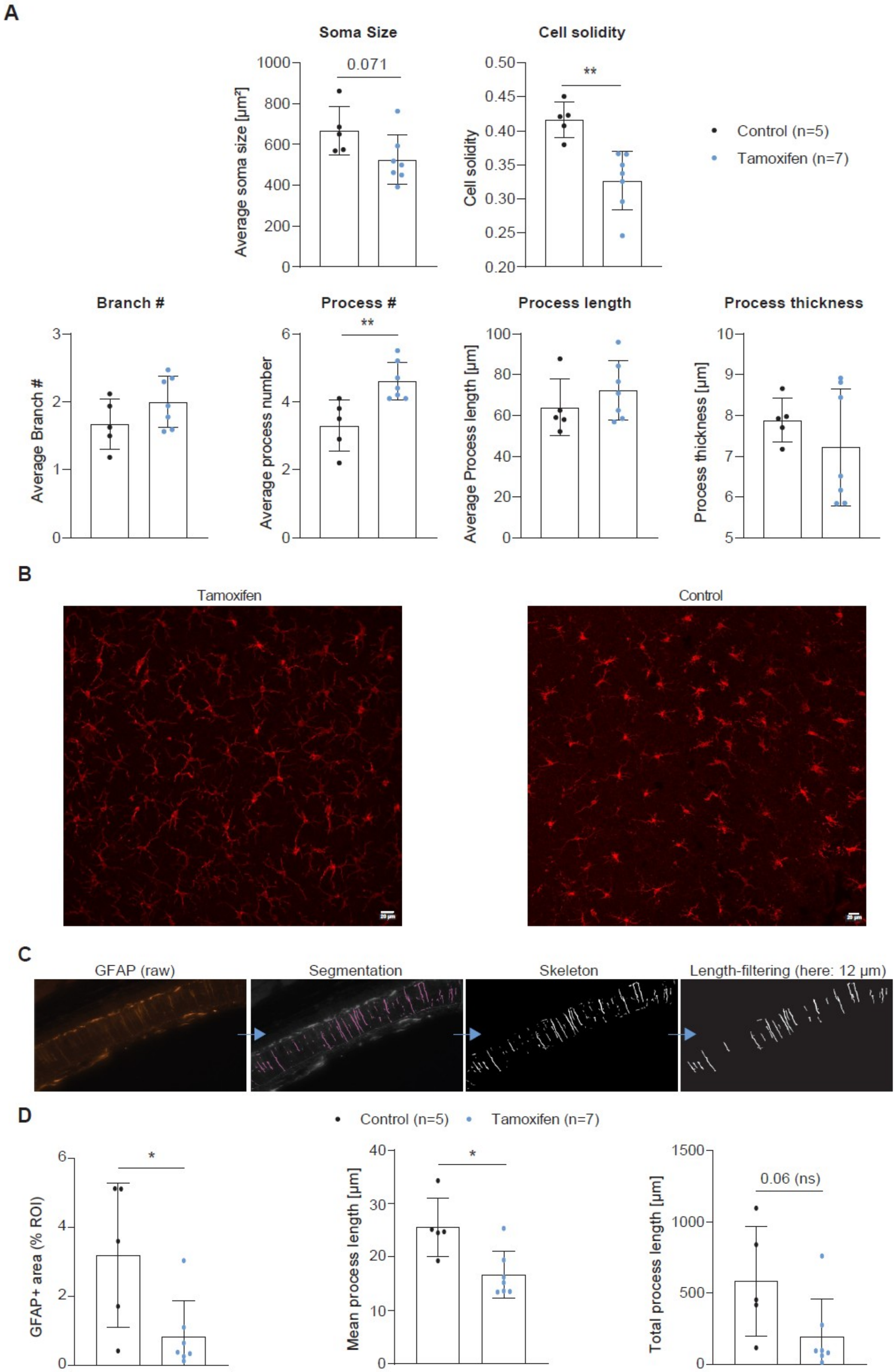

bioRxiv preprint doi: https://doi.org/10.64898/2026.09.29.753881; this version posted October 5, 2026. The copyright holder for this preprint (which was not certified by peer review) is the author/funder, who has granted bioRxiv a license to display the preprint in perpetuity. It is made available under a CC-BY 4.0 International license.

Supplementary Fig. 9. Microglial morphometry and Müller-glia reactivity in *Activ_PGC1α*-expressing hu*RHO*$^{P347L/+}$ retinae. (**A**) Quantitative morphometry of IBA1^{+} subretinal cells (control n = 5, tamoxifen n = 7): tamoxifen-injected animals show reduced cell solidity (*P* < 0.01) and an increased number of processes per cell (*P* < 0.01), consistent with a shift from amoeboid toward ramified, homeostatic morphology; soma size, branch number, process length and process thickness do not differ significantly. (**B**) Representative IBA1-stained retinal flatmounts (tamoxifen versus control). Scale bar, 20 µm. (**C**) Image-analysis workflow for GFAP+ Müller-glial process quantification in retinal cross-sections: raw GFAP signal, automated segmentation, application of the ≥12 µm Feret length filter, and the final filtered objects used for measurement. (**D**) Quantification of GFAP^{+} area (percentage of ROI), mean process length, and total process length at the 12 µm Feret cut-off (tamoxifen n = 7, control n = 5). Bars show mean ± SD with individual animal values overlaid. GFAP^{+} area (P = 0.026) and mean process length (P = 0.010) are significantly reduced in tamoxifen animals; total process length shows a non-significant reduction (P = 0.064). Unpaired two-tailed Student's t-test; *P < 0.05, ns = not significant.

bioRxiv preprint doi: https://doi.org/10.64898/2026.09.29.753881; this version posted October 5, 2026. The copyright holder for this preprint (which was not certified by peer review) is the author/funder, who has granted bioRxiv a license to display the preprint in perpetuity. It is made available under a CC-BY 4.0 International license.

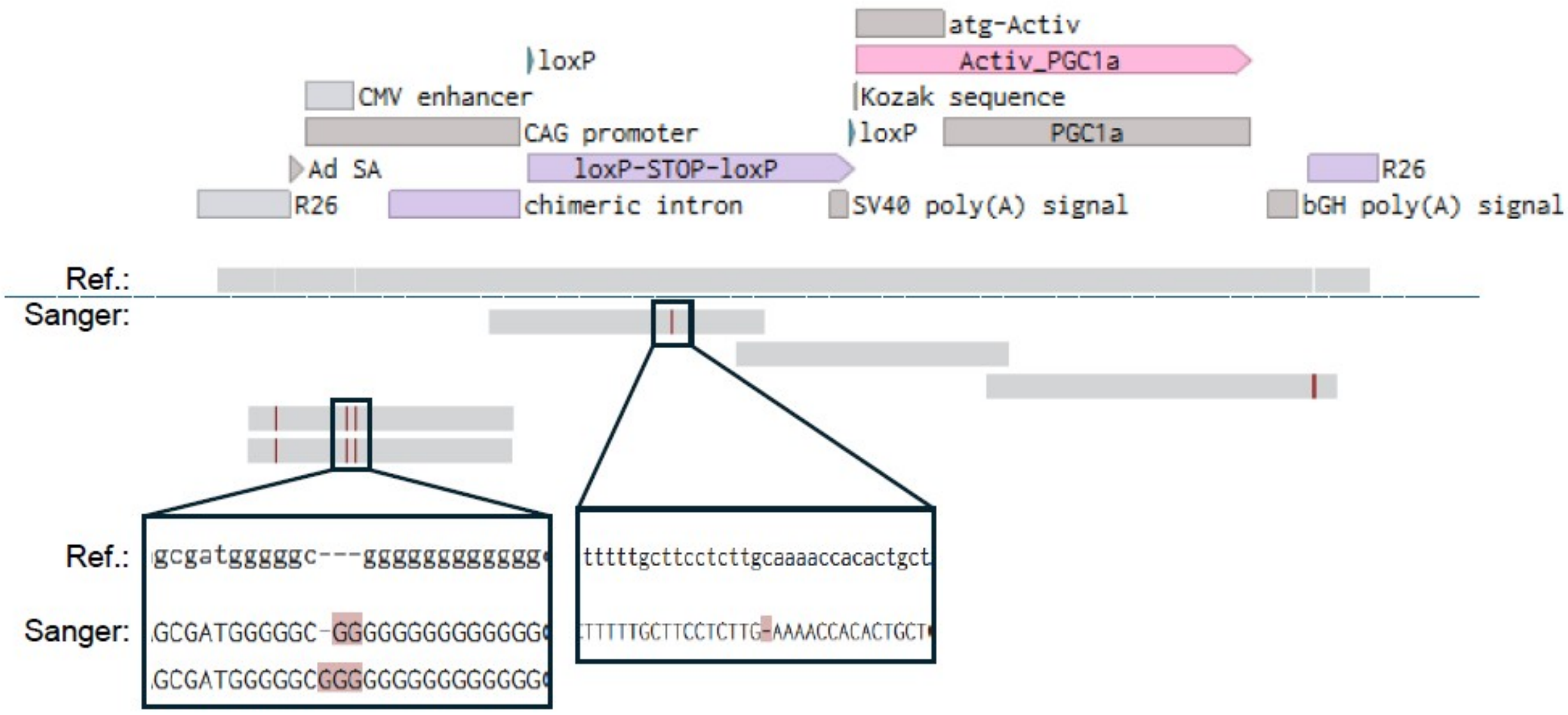

Supplementary Fig. 10. Sanger-sequencing validation of the *R26*$^{LSL\text{-}Activ\_PGC1\alpha}$ knock-in cassette. (**Top**) Annotated map of the targeted *Rosa26* allele (5′→3′): *R26* homology arm, CAG promoter (CMV enhancer, chimeric intron), loxP-flanked transcriptional STOP cassette, the *Activ_PGC1α* transgene (atg-Activ start, Kozak sequence, Activ_PGC1α–*Ppargc1a* ORF), SV40 and bGH poly(A) signals, and the distal *R26* arm. (**Middle**) Coverage of five overlapping Sanger reads tiled across the full cassette, aligned to the *in silico* targeted-allele reference. (**Bottom**) Alignment detail at the two positions that differ from the reference (red): a poly-G run in the CAG-promoter region and an A/T-rich indel within the loxP-STOP-loxP cassette. Both are Sanger homopolymer basecalling artifacts (G-tract compression and A/T-run length ambiguity), and both lie outside the Activ_PGC1α coding sequence, which matched the reference at 100% identity with no discordant bases. Reference (Ref.) and Sanger consensus (Sanger) tracks are indicated at left. Alignment was performed with Auto-MAFFT on Benchling.

bioRxiv preprint doi: https://doi.org/10.64898/2026.09.29.753881; this version posted October 5, 2026. The copyright holder for this preprint (which was not certified by peer review) is the author/funder, who has granted bioRxiv a license to display the preprint in perpetuity. It is made available under a CC-BY 4.0 International license.

Supplementary Table 1: Postmortem eye donor information

Donor	Age	Gender	Cause of death	Ocular History	Relevant Medical History
A	29 y	Female	Liver failure	Cotton wool spots (OU), scleral icterus (OU)	Type 2 diabetes mellitus, systemic lupus erythematosus
B	51 y	Female	Acute respiratory distress syndrome (ARDS)	Keratopathy, transient eyelid edema	Obesity
C	54 y	Female	Subarachnoid hemorrhage	None documented.	Hypertension, history of tobacco use

bioRxiv preprint doi: https://doi.org/10.64898/2026.09.29.753881; this version posted October 5, 2026. The copyright holder for this preprint (which was not certified by peer review) is the author/funder, who has granted bioRxiv a license to display the preprint in perpetuity. It is made available under a CC-BY 4.0 International license.

Supplementary Table 2: Activ_PGC1α coding sequence (left) and predicted amino acid sequence (right)

Coding sequence (5’ → 3’)	Predicted amino acid sequence
atgggtgggggtgggtccagcgggttgcctaatgggttggatggtgacgag<br>gattttagcgatatcgccgatatggacttttcagctctcctcagccagataagc<br>tctggaagtagtgggcttccaaatgggcttgacggtgacgaagatttctctg<br>atatagcggacatggacttctcagctctgctctcccagatttcttcagggtcca<br>gcggcttgcccaacggcctcgatggcgacgaggatttttctgacatagcag<br>acatggacttttccgcactcctcagtcagatttcttctggaggcggtggaagc<br>ggcttcagcgtggacaccagtgccctgctggacctgttcagcccctcggtg<br>accgtgcccgacatgagcctgcctgaccttgacagcagcctggccagtatc<br>caagagctcctgtctccccaggagcccccccaggcctcccgaggcagaga<br>acagcagcccggattcagggaagcagctggtgcactacacagcgcagc<br>cgctgttcctgctggaccccggctccgtggacaccgggagcaacgacctg<br>ccggtgctgtttgagctgggagagggctcctacttctccgaaggggacggc<br>ttcgccgaggaccccaccatctccctgctgacaggctcggagcctcccaa<br>agccaaggaccccactgtctccatggcttgggacatgtgcagccaagact<br>ctgtatggagtgacatagagtgtgctgctctggttggtgaggaccagcctcttt<br>gcccagatcttcctgaacttgacctttctgaacttgatgtgaatgacttggata<br>cagacagctttctgggtggattgaagtggtgtagcgaccaatcggaaatca<br>tatccaaccagtacaacaatgagcctgcgaacatatttgagaagatagatg<br>aagagaatgaggcaaacttgctagcggttctcacagagacactggacagt<br>ctccccgtggatgaagacggattgccctcatttgatgcactgacagatgga<br>gccgtgaccactgacaacgaggccagtccttcctccatgcctgacggcac<br>ccctccccctcaggaggcagaagagccgtctctacttaagaagctcttact<br>ggcaccagccaacactcagctcagctacaatgaatgcagcggtcttagca<br>ctcagaaccatgcagcaaaccacacccacaggatcagaacaaaccctg<br>ccattgttaagaccgagaattcatggagcaataaagcgaagagcatttgtc<br>aacagcaaaagccacaaagacgtccctgctcagagcttctcaagtatctg<br>accacaaacgatgaccctcctcacaccaaacccacagaaaacaggaac<br>agcagcagagacaaatgtgcttcgaaaaagaagtcccatacacaaccg<br>cagtcgcaacatgctcaagccaaaccaacaactttatctcttcctctgaccc<br>cagagtcaccaaatgaccccaagggttccccatttgagaacaagactattg<br>agcgaaccttaagtgtggaactctctggaactgcaggcctaactcctccca<br>caactcctcctcataaagccaaccaagataaccctttcaaggcttcgccaa<br>agctgaagccctcttgcaagaccgtggtgccaccgccaaccaagagggc<br>ccggtacagtgagtgttctggtacccaaggcagccactccaccaagaaag<br>ggcccgagcaatctgagttgtacgcacaactcagcaagtcctcagggctc<br>agccgaggacacgaggaaaggaagactaaacggcccagtctccggct<br>gtttggtgaccatgactactgtcagtcactcaattccaaaacggatatactca<br>ttaacatatcacaggagctccaagactctagacaactagacttcaaagatg<br>cctcctgtgactggcaggggcacatctgttcttccacagattcaggccagtg<br>ctacctgagagagactttggaggccagcaagcaggtctctccttgcagcac<br>cagaaaacagctccaagaccaggaaatccgagcggagctgaacaagc<br>acttcggtcatccctgtcaagctgtgtttgacgacaaatcagacaagaccag<br>tgaactaagggatggcgacttcagtaatgaacaattctccaaactacctgtg<br>tttataaattcaggactagccatggatggcctatttgatgacagtgaagatga<br>aagtgataaactgagctacccttgggatggcacgcagccctattcattgttc<br>gatgtgtcgccttcttgctcttcctttaactctccgtgtcgagactcagtgtcacc<br>accgaaatccttattttctcaaagaccccaaaggatgcgctctcgttcaagat<br>ccttttctcgacacaggtcgtgttcccgatcaccatattccaggtcaagatca<br>aggtccccaggcagtagatcctcttcaagatcctgttactactatgaatcaag<br>ccactacagacaccgcacacaccgcaattctcccttgtatgtgagatcacgt<br>tcaaggtcaccctacagccgtaggcccaggtacgacagctatgaagccta<br>tgagcacgaaaggctcaagagggatgaataccgcaaagagcacgaga<br>agcgggagtctgaaagggccaaacagagagagaggcagaagcagaa<br>agcaattgaagagcgccgtgtgatttacgttggtaaaatcagacctgacac<br>aacgcggacagaattgagagaccgctttgaagtttttggtgaaattgagga<br>atgcaccgtaaatctgcgggatgatggagacagctatggtttcatcacctac	MGGGGSSGLPNGLDGDEDFSDIADMDFSALLSQISS<br>GSSGLPNGLDGDEDFSDIADMDFSALLSQISSGSSGL<br>PNGLDGDEDFSDIADMDFSALLSQISSGGGGSGFSV<br>DTSALLDLFSPSVTVPDMSLPDLDSSLASIQELLSPQE<br>PPRPPEAENSSPDSGKQLVHYTAQPLFLLDPGSVDT<br>GSNDLPVLFELGEGSYFSEGDGFAEDPTISLLTGSEP<br>PKAKDPTVSMAWDMCSQDSVWSDIECAALVGEDQP<br>LCPDLPELDLSELDVNDLDTDSFLGGLKWCSDQSEIIS<br>NQYNNEPANIFEKIDEENEANLLAVLTETLDSLPVDED<br>GLPSFDALTDGAVTTDNEASPSSMPDGTPPPQEAEE<br>PSLLKKLLLAPANTQLSYNECSGLSTQNHAANHTHRI<br>RTNPAIVKTENSWSNKAKSICQQQKPQRRPCSELLKY<br>LTTNDDPPHTKPTENRNSSRDKCASKKKSHTQPQSQ<br>HAQAKPTTLSLPLTPESPNDPKGSPFENKTIERTLSVE<br>LSGTAGLTPPTTPPHKANQDNPFKASPKLKPSCKTVV<br>PPPTKRARYSECSGTQGSHSTKKGPEQSELYAQLSK<br>SSGLSRGHEERKTKRPSLRLFGDHDYCQSLNSKTDIL<br>INISQELQDSRQLDFKDASCDWQGHICSSTDSGQCYL<br>RETLEASKQVSPCSTRKQLQDQEIRAELNKHFGHPC<br>QAVFDDKSDKTSELRDGDFSNEQFSKLPVFINSGLAM<br>DGLFDDSEDESDKLSYPWDGTQPYSLFDVSPSCSSF<br>NSPCRDSVSPPKSLFSQRPQRMRSRSRSFSRHRSC<br>SRSPYSRSRSRSPGSRSSSRSCYYYESSHYRHRTHR<br>NSPLYVRSRSRSPYSRRPRYDSYEAYEHERLKRDEY<br>RKEHEKRESERAKQRERQKQKAIEERRVIYVGKIRPD<br>TTRTELRDRFEVFGEIEECTVNLRDDGDSYGFITYRYT<br>CDAFAALENGYTLRRSNETDFELYFCGRKQFFKSNY<br>ADLDTNSDDFDPASTKSKYDSLDFDSLLKEAQRSLRR<br>*

bioRxiv preprint doi: https://doi.org/10.64898/2026.09.29.753881; this version posted October 5, 2026. The copyright holder for this preprint (which was not certified by peer review) is the author/funder, who has granted bioRxiv a license to display the preprint in perpetuity. It is made available under a CC-BY 4.0 International license.

**Coding sequence (5’ → 3’)**	**Predicted amino acid sequence**
cgttacacctgtgacgctttcgctgctcttgagaatggatatactttacgcagg<br>tcgaacgaaactgacttcgagctgtacttttgtggacggaagcaatttttcaa<br>gtctaactatgcagacctagataccaactcagacgattttgaccctgcttcca<br>ccaagagcaagtatgactctctggattttgatagtttactgaaggaagctca<br>gagaagcttgcgcaggtag	

bioRxiv preprint doi: https://doi.org/10.64898/2026.09.29.753881; this version posted October 5, 2026. The copyright holder for this preprint (which was not certified by peer review) is the author/funder, who has granted bioRxiv a license to display the preprint in perpetuity. It is made available under a CC-BY 4.0 International license.

Supplementary Table 3: Quantitative PCR primers

Species	Gene	Role	Forward primer (5'→3')	Reverse primer (5'→3')
Human	TNFα	Target	GCCCAGGCAGTCAGATCATC	TATCTCTCAGCTCCACGCCA
Human	IL-6	Target	CAGTTCCTGCAGAAAAAGGCAA	AGCTGCGCAGAATGAGATGA
Human	ACTB	Reference	ACCTTCTACAATGAGCTGCG	CTGGATGGCTACGTACATGG
Mouse	Tnfα	Target	CCCTCACACTCAGATCATCTTCT	TGAAGAGAACCTGGGAGTAGACA
Mouse (and human)	Pgc1α	Target	AAAGCAATTGAAGAGCGCCG	TAGCTGTCTCCATCATCCCG
Mouse	Rps18	Reference	GGATGTGAAGGATGGGAAGT	CCCTCTATGGGCTCGAATTT
